# Cardiac Sarcoma: A Rare Etiology of Outflow Tract Obstruction

**DOI:** 10.1016/j.atssr.2026.03.018

**Published:** 2026-04-17

**Authors:** Ioana B. Florea, Sajjad Sabir, Jeffrey Lee, Sarah Van Dyke, Gary Cook, Michael Rosenbloom, David D. Shersher, Kenji Minakata

**Affiliations:** 1 Department of Surgery, Cooper University Hospital, Camden, New Jersey; 2 Division of Cardiology, Department of Medicine, Cooper University Hospital, Camden, New Jersey; 3 Division of Cardiac Surgery, Department of Surgery, Cooper University Hospital, Camden, New Jersey; 4 Cooper Medical School of Rowan University, Camden, New Jersey

## Abstract

Cardiac sarcomas are exceedingly rare clinical entities that can require expeditious surgical intervention for relief of symptomatic and hemodynamic sequelae and for improved oncologic outcomes. Here we present 2 cases of primary sarcoma resulting in markedly symptomatic obstruction of the left and right ventricular outflow tracts, thus highlighting the principles of surgical intervention for these rare malignant lesions.

Primary cardiac tumors are exceedingly rare, with an autopsy incidence of 0.001% to 0.03%, and 25% of these tumors are malignant.^1^ Sarcoma histologic subtypes constitute 75% of these primary malignant lesions. On initial imaging, they may mimic alternative, more common diseases such as endocarditis or pulmonary thrombus, and thus appropriate diagnosis can be delayed.

## Case Reports

These cases represent 2 cardiac sarcomas resulting in markedly symptomatic outflow tract obstructions mandating prompt evaluation and surgical management.

### Patient 1

**Figure 1:**
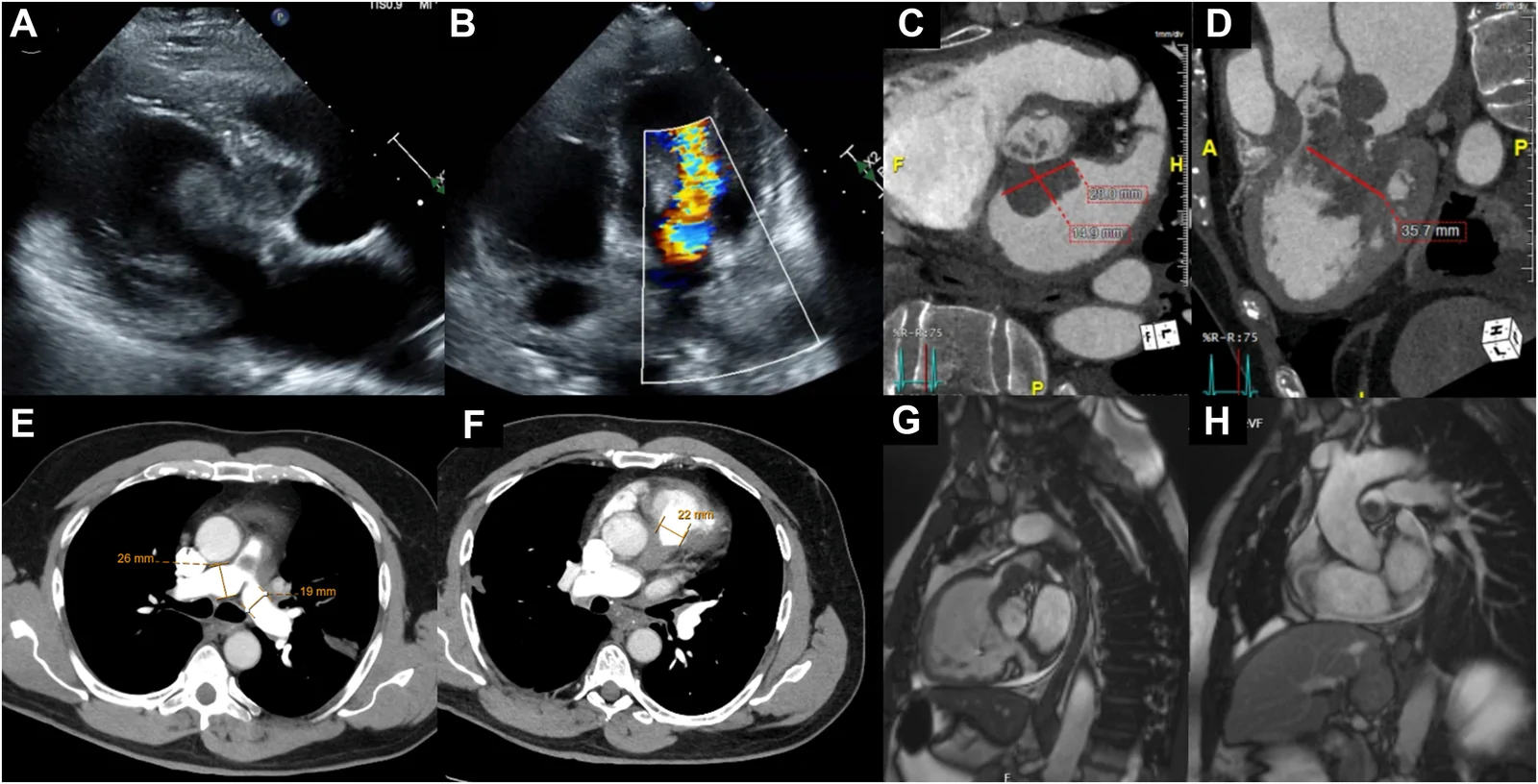
Preoperative imaging. (A-D) Patient 1. (A) Transthoracic echocardiogram demonstrating a large mass at the anterior mitral leaflet that extended into the left ventricular outflow tract. (B) Prolapse of the mass across the aortic valve during systole with severe functional mitral stenosis. (C, D) Computed tomography revealing a 36 mm × 34 mm × 18 mm heterogenous, multilobulated mass attached to the aortomitral curtain and posteromedial papillary muscle and resulting in nearly complete left ventricular outflow tract obstruction. (E-H) Patient 2. (E, F) Computed tomography demonstrating a 2.5 cm × 1.9 cm soft tissue mass along the right ventricular outflow tract and resulting in severe stenosis. (G, H) Magnetic resonance imaging showing a 17 mm × 22 mm heterogenous mass in the main pulmonary artery and extending into the proximal right pulmonary artery with obstruction.

A 71-year-old woman with a history of hypothyroidism presented with several months of progressively worsening dyspnea on exertion. Transthoracic echocardiography (TTE) identified a very large, mobile mass attached to the anterior leaflet of the mitral valve (MV),. The mass extended into the left ventricular outflow tract (LVOT) and resulted in severe functional mitral stenosis and a mean transmitral gradient of 18 mm Hg (Figures 1A, 1B). A comprehensive infectious workup did not reveal acute endocarditis. Further evaluation with cardiac computed tomography demonstrated a heterogenous, multilobulated large mass causing nearly complete LVOT obstruction (Figures 1C, 1D; Video 1).

**Figure 2:**
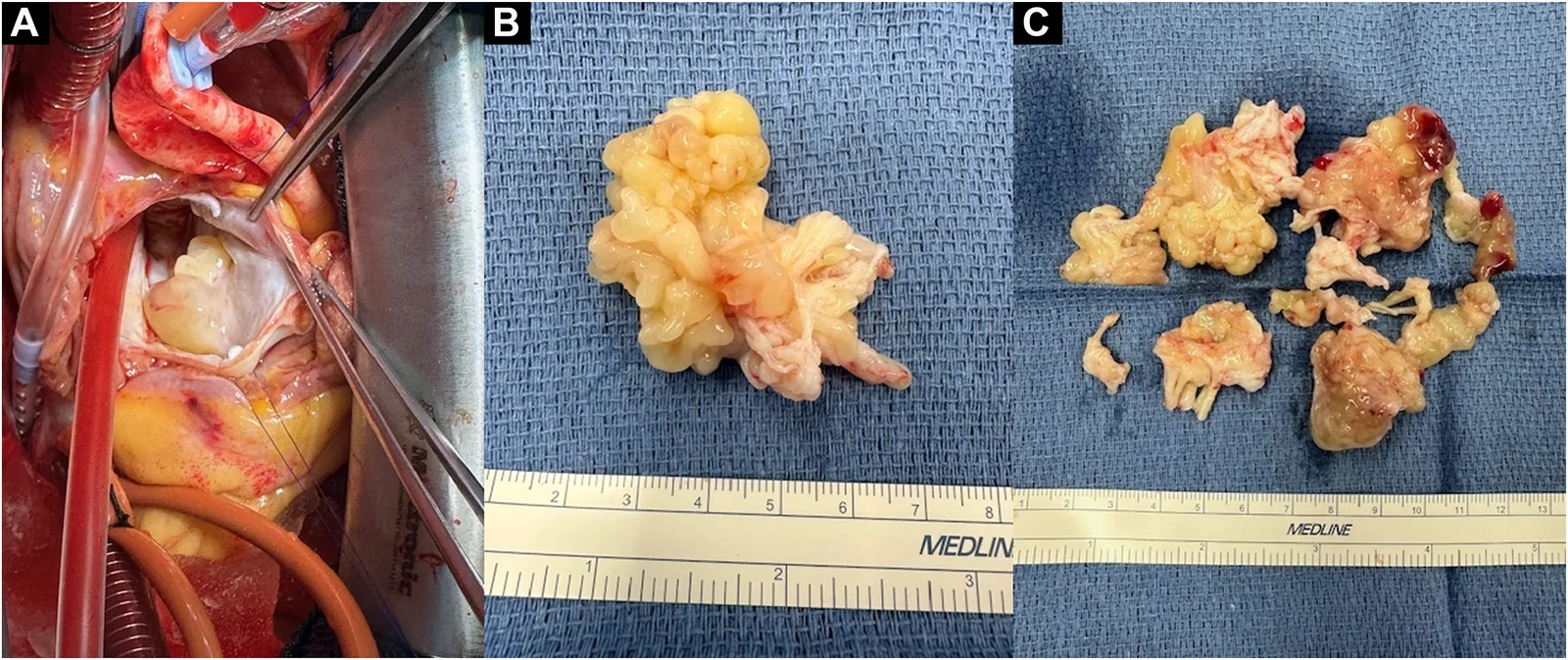
Patient 1: intraoperative findings. (A) Mass along the anterior mitral valve leaflet with extension into the left ventricular outflow tract. (B) High-grade intimal sarcoma. (C) Mass alongside resected fragments of the mitral leaflet and extension into the left ventricle and papillary muscles.

The patient was subsequently taken to the operating room for urgent tumor resection. Standard cardiopulmonary bypass (CPB) was achieved with aortic and bicaval cannulations, and Del Nido cardioplegia was administered under aortic cross-clamping. A superior transseptal approach permitted excellent MV exposure. A large mass was identified covering the anterior leaflet of the MV and extending into the left atrial wall (Figure 2A). The anterior leaflet appeared thickened with shortened chordal structures, and it was resected in its entirety along with the mass (Figure 2B); the posterior leaflet exhibited degenerative changes and required further resection. On close inspection, it then became apparent that there was greater extension of the mass into the left ventricular (LV) side involving the papillary muscle. This was also resected alongside the tip of the papillary muscle (Figure 2C). There was no evidence of aortic valve involvement. The MV was then replaced with a 27-mm bovine pericardial valve. Transesophageal echocardiography (TEE) at completion of the case demonstrated a normally functioning MV prothesis without a paravalvular leak, as well as preserved LV systolic function and mild aortic regurgitation. She was subsequently weaned from CPB without difficulty. Total CPB time and aortic cross-clamp time were 93 minutes and 78 minutes, respectively.

The patient’s postoperative course was unremarkable. The final pathology report revealed a high-grade intimal sarcoma. Positron emission tomography/computed tomography demonstrated a pancreatic tail lesion and scattered hypermetabolic masses in the iliac bone and femur. Biopsy of this pancreatic mass revealed a high-grade spindle cell sarcoma, consistent with metastasis. She has since initiated chemotherapy with doxorubicin (Adriamycin), ifosfamide, and mesna. On surgical follow-up, she has recovered well, with no postoperative cardiac complications. She is doing well from a cardiac standpoint, with New York Heart Association functional class II at the last follow-up 13 months postoperatively.

### Patient 2

A 60 year-old man with a history of hypertension was admitted to our institution (Cooper University Hospital, Camden, NJ) with severe right ventricular (RV) dysfunction in the setting of a newly diagnosed anterior mediastinal soft tissue mass. TTE revealed severely reduced RV systolic function (RV systolic pressure, 88 mm Hg), RV dilation with flattening of the intraventricular septum, severe tricuspid regurgitation (TR), and severe pulmonic stenosis. Diagnostic imaging demonstrated a 2.5-cm mediastinal soft tissue mass concerning for malignancy in the main pulmonary artery (PA) and extending into the proximal right PA with adjacent flow acceleration suggestive of right ventricular outflow tract (RVOT) obstruction (Figures 1E-1H; Videos 2, 3). Right-sided heart catheterization revealed PA pressure of 25/14 mm Hg (mean 19 mm Hg), pulmonary capillary wedge pressure of 8 mm Hg, mixed venous saturation of 63.1%, and a peak gradient across the pulmonic valve of 69 mm Hg.

Multidisciplinary discussions were held regarding the patient’s options for tissue diagnosis and subsequent management. The primary mediastinal soft tissue mass was itself not amenable to bronchoscopic or radiologic biopsy. Interventional pulmonology further deemed the patient too high risk to undergo robotic bronchoscopy with general anesthesia in the setting of his severe RV dysfunction and RVOT obstruction.

**Figure 3:**
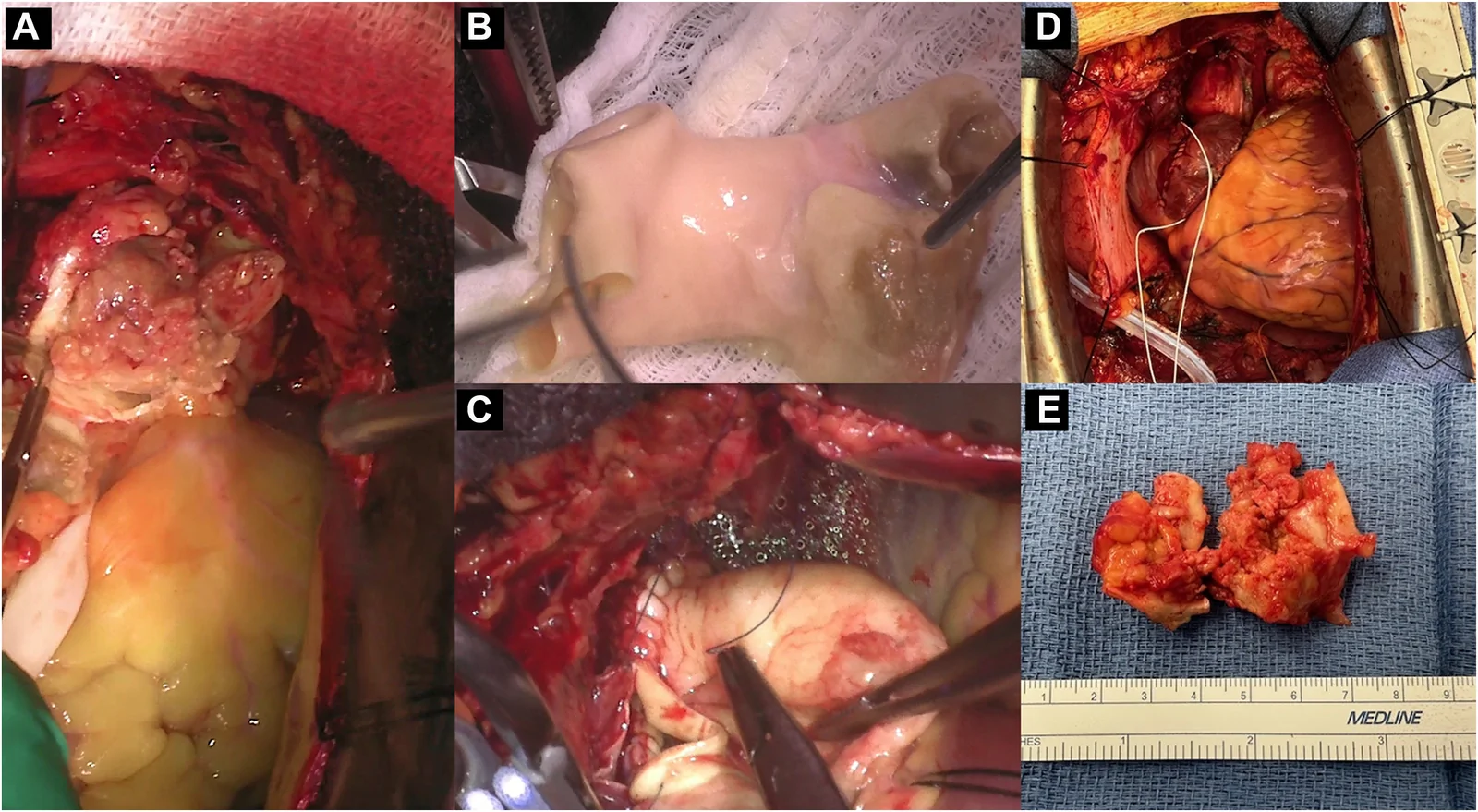
Patient 2: intraoperative findings. (A) Dissection of a mass along the intima of the pulmonary trunk. (B, C) Reconstruction of the right ventricular outflow tract and pulmonic valve with a pulmonary homograft. (D) Completed right ventricular outflow tract reconstruction. (E) Undifferentiated pleomorphic sarcoma.

Given the patient’s progressively worsening symptomatic status and limited options for alternative diagnostic testing, he was offered surgical resection. Intraoperative TEE confirmed marked RV dilation, TR, and an obstructing lesion at the pulmonic outflow tract (Video 4). CPB was instituted with aortic and bicaval cannulations, and Del Nido cardioplegia was delivered into the aortic root under aortic cross-clamping; circulatory arrest was not required. The main PA was exposed, and a longitudinal incision was made extending distally to the bifurcation into the left and the right PA, thus revealing the tumor and severe stenosis of the main PA lumen. The mass was dissected from the intima of the main PA and removed from both the left and the right main PA, with some degree of pulmonary endarterectomy (Figures 3A, 3E; Video 5). Attention was then turned to the proximal PA. The RVOT was severely stenosed, and the pulmonic valve essentially obliterated by the tumor. This required complete resection of the proximal pulmonary trunk and reconstruction of the RVOT with a 26-mm pulmonary homograft (Figures 3B-3D). TEE at the conclusion of the case revealed preserved LV systolic function and improved TR. The patient was subsequently weaned from CPB without difficulty.

The patient’s postoperative course was uncomplicated. Postoperative TTE revealed improved but persistent moderate enlargement of the right ventricle with moderately reduced RV systolic function and mildly elevated PA systolic pressure estimated at 43 mm Hg. Pathologic findings were consistent with an R1 resection of a high-grade, undifferentiated pleomorphic sarcoma. He is currently undergoing chemotherapy and radiation. At the time of his office visit 10 months postoperatively, he continues to do well from a cardiac standpoint, with New York Heart Association functional class II.

## Comment

Primary cardiac tumors are aggressive oncologic entities and carry a high burden of morbidity despite their clinical rarity. Presentation may be delayed because many patients exhibit symptomatic sequelae only after local invasion, and the diagnosis may be confounded by more common differential diagnoses. A 2020 analysis of 442 patients with primary cardiac sarcomas in the Surveillance, Epidemiology, and End Results (SEER) database revealed a median survival of 7 months and a 5-year survival of 9.8%; surgery and chemotherapy were both independently associated with increased median survival.^2^ These lesions thus benefit from expeditious workup and surgical intervention.

Given the rarity of these tumors, no standardized treatment paradigms exist for approaching such masses. Thus, multidisciplinary institutional approaches at high-volume centers are key for guiding therapy. The general principle of surgical intervention is to obtain an R0 resection where safe and anatomically feasible; early diagnosis of a malignant tumor and the use of neoadjuvant therapy should be considered to facilitate this objective.^1^^,^^3^

These 2 cases represent patients whose cardiac masses were markedly symptomatic secondary to outflow tract obstructions, thus necessitating upfront surgical management. These cases highlight our approaches toward exposure of the LVOT and the RVOT, as well as the necessity of exploring for mass extension into adjacent structures that may require further surgical intervention to achieve complete oncologic resection. Patients can have excellent postoperative functional outcomes and can transition toward systemic chemotherapy as indicated.

## References

[1] Ramlwai B., Leja M.J., Abu Saleh W.K. (2016). Surgical treatment of primary cardiac sarcomas: review of a single-institution experience. Ann Thorac Surg.

[2] Yin K., Luo R., Wei Y. (2021). Survival outcomes in patients with primary cardiac sarcoma in the United States. J Thorac Cardiovasc Surg.

[3] Li H., Xu D., Chen Z. (2014). Prognostic analysis for survival after resections of localized primary cardiac sarcomas: a single-institution experience. Ann Thorac Surg.

